# Parenteral Lipid-Based Nanoparticles for CNS Disorders: Integrating Various Facets of Preclinical Evaluation towards More Effective Clinical Translation

**DOI:** 10.3390/pharmaceutics15020443

**Published:** 2023-01-29

**Authors:** Tanja Ilić, Jelena B. Đoković, Ines Nikolić, Jelena R. Mitrović, Ivana Pantelić, Snežana D. Savić, Miroslav M. Savić

**Affiliations:** 1 Department of Pharmaceutical Technology and Cosmetology, University of Belgrade-Faculty of Pharmacy, Vojvode Stepe 450, 11221 Belgrade, Serbia; 2 Department of Pharmacology, University of Belgrade-Faculty of Pharmacy, Vojvode Stepe 450, 11221 Belgrade, Serbia

**Keywords:** liposomes, nanoemulsions, solid lipid nanoparticles, nanostructured lipid carriers, blood–brain barrier, brain targeting ligands, critical quality attributes, pharmacokinetic and biodistribution studies

## Abstract

Contemporary trends in combinatorial chemistry and the design of pharmaceuticals targeting brain disorders have favored the development of drug candidates with increased lipophilicity and poorer water solubility, with the expected improvement in delivery across the blood–brain barrier (BBB). The growing availability of innovative excipients/ligands allowing improved brain targeting and controlled drug release makes the lipid nanocarriers a reasonable choice to overcome the factors impeding drug delivery through the BBB. However, a wide variety of methods, study designs and experimental conditions utilized in the literature hinder their systematic comparison, and thus slows the advances in brain-targeting by lipid-based nanoparticles. This review provides an overview of the methods most commonly utilized during the preclinical testing of liposomes, nanoemulsions, solid lipid nanoparticles and nanostructured lipid carriers intended for the treatment of various CNS disorders via the parenteral route. In order to fully elucidate the structure, stability, safety profiles, biodistribution, metabolism, pharmacokinetics and immunological effects of such lipid-based nanoparticles, a transdisciplinary approach to preclinical characterization is mandatory, covering a comprehensive set of physical, chemical, in vitro and in vivo biological testing.

## 1. Introduction

Despite substantial progress achieved in neuroscience and neuropsychopharmacology, effective treatment of various neurodegenerative, cerebrovascular and psychiatric disorders, as well as infections of the central nervous system (CNS), is still an unsolved challenge. Conventional dosage forms are associated with a lack of targetability, which may contribute to low therapeutic concentrations within the brain and, consequently, the suboptimal therapeutic outcome [1].

Approximately 98% of currently available drugs were estimated as ineffective for the treatment of CNS pathologies due to their inability to efficiently overcome the highly restrictive blood–brain barrier (BBB) after systemic administration [2,3]. The unique structure of BBB, characterized by tightly connected endothelial capillary cells that are surrounded by the pericytes, basal lamina and astrocytic perivascular endfeet, is crucial for maintaining the brain’s homeostasis and protection against pathogens and noxious chemicals. Owing to (i) the presence of tight junctions (brain endothelial cells are 50–100 times tighter than normal circulation endothelial cells), (ii) intensive metabolic activity (endothelial cells express a number of ectoenzymes, such as aminopeptidases, endopeptidases and cholinesterases), and (iii) expression of ion channels and the influx/efflux transporters, the BBB controls and limits translocation of drugs and other molecules to the brain [2,4,5,6]. Generally, only small (molecular weight lower than 500 Da), lipophilic (high partition coefficient) and non-ionized drugs, with less than 8–10 hydrogen bonds, can easily diffuse across the BBB by a simple transmembrane diffusion. Therefore, it is obvious that these properties deter most drugs from entering the brain at therapeutically effective levels [2,7]. As a result, the contemporary trends in combinatorial chemistry and the design of pharmaceuticals targeting brain disorders have favored the development of drug candidates with greater lipophilicity and consequently, poorer water solubility, with the expected increase in BBB delivery. However, for poorly soluble substances, parenteral administration is not conceivable without using high concentrations of organic solvents and/or surfactants, thus increasing the likelihood of vehicle-related safety issues [8]. Among the various strategies studied to overcome the poor water solubility of various CNS-active drugs as well as the hurdles in BBB crossing, lipid-based nanoparticles (LNs) have been recognized as an excellent platform for brain targeting.

Liposomes, nanoemulsions, solid lipid nanoparticles and nanostructured lipid carriers, as the most important representatives of LNs, have been used as carriers for poorly water-soluble small molecule drugs for over 30 years [9]. However, the growing availability of innovative excipients/ligands allowing improved brain targeting ability and controlled drug release, thrust the lipid nanocarriers back to the limelight for overcoming the factors that impede drug delivery through the BBB. Likewise, it is interesting to note that, concomitantly with advances in the development of synthetic lipid-based nanoparticles, in recent years, exosomes—nanosized lipid-based vesicles secreted by different living cells, including neurons, microglia and astrocytes—have emerged as promising vehicles for brain-targeted drug delivery. Although the role of exosomes in the pathogenesis of various neurological disorders has not yet been fully elucidated, due to their natural ability to cross the BBB and carry the drugs with different physicochemical properties, as well as surface modification flexibility enabling specific brain cell targeting, exosomes have been increasingly used to deliver drugs in various brain disorders (for more details on exosomes for brain drug delivery, their advantages and limitations, please see [10,11,12]. On the other hand, owing to easily tunable properties, lipid-based nanoparticles offer numerous appealing features compared to polymeric or inorganic nanoparticles, such as (1) biodegradability, biocompatibility, lower toxicity and immunogenicity, (2) potential for encapsulation of both hydrophilic and lipophilic drugs, (3) higher loading capacity for hydrophobic drugs and higher control over drug release kinetics, (4) protection of drugs from undesirable hydrolytic and enzymatic degradation, (5) easier manufacturing and cost-effective large scale production. In other words, the key benefit of drug loading into the LNs for brain delivery is the capability to improve the pharmacokinetic profile providing higher concentrations in the brain parenchyma of drugs that exhibit poor brain disposition. Precisely, due to lipophilic nature and small size, LNs have a natural tendency to cross the BBB without any functionalization. In addition, the surface modifications of LNs with hydrophilic polymers, surfactants, peptides and other coating agents can significantly improve the amount of drug that reaches the brain parenchyma, directly, via the interactions of LN with receptors/target transporters on the BBB and/or indirectly, via reduced recognition and clearance of LN by reticulo-endothelial system (RES) and prolonged circulatory time [2,5,13]. However, it should be emphasized that, to this date, despite the substantial achievements in the brain-targeted drug delivery area, only a small number of these systems entered clinical trials or became commercially available.

**Figure 1:**
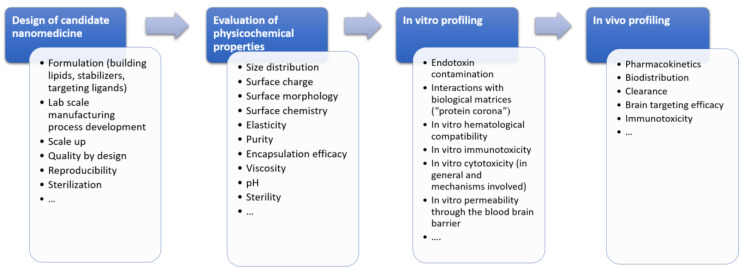
Key areas in the preclinical development of parenteral lipid-based nanoparticles specifically intended for neurological and other CNS disorders. The list is not exhaustive, as indicated by the last bullets with “…” following every list.

Many challenges have been identified as bottlenecks during the development and preclinical testing of lipid-based nanoformulations for brain-targeting that hamper their translation from the discovery-phase to clinical trials. The first critical hurdle encompasses the design of a suitable formulation that is capable to (i) transport a sufficient payload of the therapeutic agent, without premature leakage or off-target release, and to (ii) reach the efficient concentration at the target site within the brain, overcoming multiple biological barriers (opsonization and clearance by reticulo-endothelial system (RES), the BBB) [14]. During recent years, various ligands that either specifically bind to the receptors/target transporters overexpressed on the brain endothelial cells or impede recognition by RES have been utilized to achieve this goal. Traditional nanocarrier development has been based on a formulation-driven approach, engineered and characterized from a physicochemical perspective, without a comprehensive biological proof of concept [14]. However, this has imposed numerous concerns regarding quality, safety and efficacy of these sophisticated LNs, including the reproducibility of the manufacturing process and adaptation to large-scale production [15,16]. Therefore, a rational design of innovative LNs for optimized brain disposition requires integrated in chemico/in vitro/in vivo multiparameter-testing approach, summarized in Figure 1.

Despite a general trend towards nanomedicine development and application, there is a scarcity of specific and standardized protocols applicable for the characterization of complex and innovative nanoformulations, at both physicochemical and biological levels [17]. Hence, the identification of appropriate methodologies from a technical and regulatory standpoint, as well as the lack of uniformity in various preclinical assays, has been recognized as major challenges hampering the successful development and translation of any nanocarrier [18,19]. Moreover, a wide variety of methods, study designs and experimental conditions utilized in the literature prevent their systemic comparison and limit the advances in brain-targeting by lipid-based nanoparticles. Therefore, this review intends to provide an overview of the most commonly utilized methods during the preclinical testing of lipid-based nanoparticles (liposomes, nanoemulsions, solid lipid nanoparticles and nanostructured lipid carriers) intended for the treatment of various CNS disorders via the parenteral route. Particular attention was given to the methodologies applied for formulation design and manufacturing process optimization, along with (i) the assessment of the critical quality attributes, (ii) in vitro assays for early screening of the nanocarrier safety (i.e., in vitro cytotoxicity and hemocompatibility tests, as most frequently reported), stability in biological surroundings and in vitro permeability through the BBB as well as (iii) in vivo methods for studying the nanocarrier disposition, with a particular emphasis on their brain targeting efficiency. The strengths and limitations of each methodology are presented in brief, pointing out some gaps and offering directions for further improvement and standardization.

## 2. Formulation-Related Considerations

The intended administration route is one of the most important factors to consider when developing a drug-delivery system (DDS). Several routes, such as parenteral, intranasal or local delivery via injection to the target site, could be considered for brain-targeting drugs [20]. Local delivery involves injecting the drug formulation directly to the target site in the brain and, while very effective in animal preclinical trials, leads to certain clinical failures due to high invasiveness, rapid drug degradation and clearance. Intranasal administration, praised for its ability to deliver drugs directly to the brain while bypassing the BBB, is highly dependent on the nature of the nasal mucosa and potential drug interactions [20]. Despite being described as invasive and painful, parenteral administration is still the most acceptable method of delivering drugs to the brain. It is the preferred route of administration in cases of emergency [21], with high bioavailability [22]. The BBB is the main obstacle to successful parenteral delivery of actives to the brain. As a result, it is critical to design delivery systems capable of overcoming this barrier and allowing the encapsulated drug to successfully reach its target site.

To select DDS with favorable properties for parenteral administration and targeted brain delivery, special attention must be paid to formulation factors in order to avoid any issues with drug targeting, such as DDS toxicity, premature release or unfavorable interactions with biological fluid components [23]. Given the toxicity of some polymeric nanoparticles, the focus of research has shifted to lipid-based nanocarriers, such as nanoemulsions, liposomes or nanoparticles, which exhibit good biocompatibility, biodegradability, and non-immunogenic properties [24]. These carriers can also be modified to achieve targeted drug delivery and improve pharmacokinetic properties. When DDS is administered parenterally, the first barrier to successful drug delivery relates to the plasma proteins that form the corona around the particles/droplets, thus determining systematic circulation time and biodistribution [25].

Liposomes are one of the most commonly used carriers for brain delivery [26]. They are made up of phospholipid bilayers that encapsulate an aqueous core and can transport both hydrophilic and lipophilic drugs, which is their main advantage over other lipid nanocarriers [23]. The primary building blocks for liposomes are phosphatidylcholines derived from soybeans or eggs, with an established impact on brain delivery [27,28,29]. Additionally, cholesterol is typically added to increase membrane rigidity and prevent payload leakage [27,28,29,30,31,32]. The addition of surfactants, such as polysorbate 80 improves entrapment stability compared to the surfactant-free vesicles [32]. Cationic lipids, such as DDAB (Table 1), can also be used in liposome preparation because electrostatic interactions between cationic liposomes and negatively charged cell membranes (specifically endothelial cells of microvessels rich in lecithin) improve particle uptake via endocytosis [32].

Nanoemulsions (NEs) for parenteral application are typically defined as oil in water systems in which oil droplets are dispersed in an aqueous system and stabilized with one or more surfactants. A drug’s solubility in oils is the driving force in the selection of the NEs’ components [33]. For parenteral application, oil cores are usually composed of soybean oil, sunflower seed oil, olive oil, palm kernel oil esters, medium-chain triglycerides or fish oil [21,34,35,36,37,38,39]. It is advised to combine oils high in long-chain triglycerides, such as soybean or sunflower oil, which may improve drug penetration across the BBB due to linoleic acid content, with oils high in medium-chain triglycerides, having a higher solvent capacity and lower viscosity [39,40]. Because some of the free fatty acids from the selected oils, particularly oleic acid, can act as co-emulsifiers, the choice of the oil phase is critical not only for adequate drug loading but also for system stabilization [41]. Natural lecithins are the most widely used stabilizers in parenteral NEs because they are among the safest emulsifiers on the market, with regulatory GRAS status. Other co-stabilizers, in addition to lecithin, can be used to improve formulation stability or achieve brain targeting (Table 1). The aqueous phase ingredients are chosen to complement the oil phase and ensure optimal pH and osmolality values, depending on the administration route, as well as the properties of the incorporated drug [42].

Depending on the content of the nanoparticle core, lipid nanoparticles are classified as solid lipid nanoparticles (SLN) or nanostructured lipid carriers (NLC). The SLN core is made up entirely of solid lipids, whereas the NLC uses a mixture of solid and liquid lipids and overcomes some of the limitations of SLN by improving loading efficiency and stability, as well as by preventing drug expulsion during storage [24]. Cetyl palmitate, glyceryl monostearate, stearic acid and glyceryl distearate are the most commonly used solid lipids, while medium-chain triglycerides are the most commonly used liquid lipids [29,43,44,45,46,47,48,49,50]. Several surfactants, such as polysorbate 60, polysorbate 80, poloxamer 188 or lecithin, could be used for additional stabilization [45,46,47,48,49,51,52]. To improve brain distribution, lipid nanoparticles can be functionalized with various ligands, as shown in Table 1.

DDS can be surface modified with various ligands to achieve successful brain delivery (Table 1). One of the major disadvantages of all nano-DDS is the rapid clearance of plasma due to the activity of the mononuclear phagocytic system (MPS). To allow the drug more time to reach the target site (in the case of brain-targeted delivery via parenteral route–BBB), one of the most effective modifications is the coating or conjugation of polyethylene glycol (PEG) on the surface of the nanocarrier, known as PEGylation [53]. This is also known as passive targeting because it gives the DDS more time to circulate and eventually reach the BBB but without a specific target. Active targeting, on the other hand, necessitates the attachment of ligands such as peptides, antibodies, or aptamers (Table 1) that can bind to specific BBB targets. These two strategies could be combined to achieve both longer circulation and target-specific binding, but special consideration should be given to the PEG chains covering the target site binding ligands. This could be overcome by using PEG derivatives as spacers between the surface of the nanoparticles and the ligands [53].

**Table 1:** Brain-targeting ligands in lipid nanoparticles.

BBB Crossing Enhancer	Drugs	Mechanism of Action	Additional Notes	References
**Liposomes**				
Glutathione and PEG2000-DSPE ***	methotrexate	Target glutathione transporter, preferentially expressed at the BBB	Brain targeting effects depend on the main phospholipid choice	[27]
Peptides derived from APP ^#^ linked to diglycerol succinate	dopamine	Target BBB transporters/receptors		[30]
Glycolipids (C12-alkyl-mannopyranoside)	dynantin	Binding to the mannose receptors (transmembrane glycoproteins)		[31]
Cationic lipids (DDAB ^##^)	andrographolide	Crossing the BBB through absorption-mediated transcytosis; possibly due to the electrostatic interactions with the negatively charged cell membranes	Could be used alone or in a combination with solubilizers	[32]
Peptide RVG29	N-3,4-bis(pivaloyloxy)-dopamine	Specifically binds to the acetylcholine receptor (AchR) on brain capillary endothelial cells and dopaminergic cells	The peptide binds to the liposome surface via coupling reaction between PEG2000-DSPE-maleimide and thiol	[54]
Aptamers (TfRA15T)	obidoxime	Binding with the transferrin receptor	Modification with the 3′-inverted deoxythymidine improves serum stability	[29]
**Nanoemulsions**				
Polysorbate 80	risperidone, cefuroxime, chloramphenicol, aripiprazole, valproic acid	Increases drug uptake through adsorption-mediated endocytosis via low-density lipoprotein receptor of brain endothelial cells; Inhibits P-glycoprotein		[21,34,37,38,39,55]
Lactoferrin (Lf) (cationic iron-binding protein) and its modifications—thiolated lactoferrin conjugate with mPEG5000-MAL **	indinavir, tanshinone I	Receptors at the BBB level allow lactoferrin to enter the brain through endocytosis	It is necessary to have a source of carboxylic acid groups at droplet interface to allow for the attachment of lactoferrin on the surface of the droplet	[36,56]
PEGylated phospholipids (PEG2000-DSPE ***; PEG5000-DPPE ****)	indinavir; curcumin	Allows for passive brain accumulation by ensuring slower plasma elimination		[40,57]
**Solid lipid nanoparticles and nanostructured lipid carriers**				
Triphenylphosphine (TPP) and RVG29	resveratrol	Binds to the acetylcholine brain receptors (RVG29) and brain mitochondria (TPP)	In order to circumvent the use of organic solvents, these ligands were attached to the mPEG-DSPE	[43]
Transferrin (Tf)	rapamycin, curcumin, quercetin	TF binds to the transferrin receptors on the surface of the endothelial cells	Carboxyl group of the Tf links to the NH2 group of the stearylamine, DSPE, PEG-NH2	[46,47,51]
OX26 conjugated with 3beta-[N-(N0, N0-dimethylaminoethane) carbamoyl] cholesterol as cationic lipid	baicalin	OX26 monoclonal antibody targets the transferrin receptor	OX26 was conjugated on the surface of the PEGylated cationic solid lipid nanoparticles	[58,59]
RVG29	quercetin	Binds to the nicotinic acetylcholine receptor expressed in the brain endothelial cells at the BBB	Thiol groups of the RVG29 react to the maleimide groups of the DSPE-PEG-MAL	[60]
Lf	riluzole; nimodipine	Binds to the Lf receptors expressed on the brain endothelial cells	Lf binds to the surface of the NLC via stearic acid/DSPE-PEG2000- COOH	[48,50]
PEG-SA	tanshinol borneol ester	PEGylation allows for longer plasma retention and subsequent higher brain concentrations compared to the non-PEGylated NLC		[49]

Abbreviations: ^#^ amyloid precursor protein; ^##^ Didecyldimethylammonium bromide; ** Methoxy polyethylene glycol 5000 maleimide; *** N-(Carbonyl-methoxypolyethylenglycol-2000)-1,2-distearoyl-sn-glycero-3-phosphoethanolamine, sodium salt); **** (N-(Carbonyl-methoxypolyethylenglycol-5000)-1,2-dipalmitoyl-sn-glycero-3-phosphoethanolamine, sodium salt.

Several techniques could be used to prepare lipid nanoparticles, including high-pressure homogenization (HPH) [25,34,36,38,40,44], thin film hydration followed by the sonication or filtration of liposomes [28,29,30,54], a combination of homogenization and sonication [46,47] or solvent-emulsification and evaporation techniques [45]. The physical and chemical complexity of the laboratory-scale prepared formulations is one of the facets that affect the scale-up of lipid nanoparticles, leading to difficulties in ensuring good stability during the shelf life [25]. Several requirements must be met for a formulation to enter the pharmaceutical market, including affordable, large-scale production techniques that meet regulatory standards. HPH is the most industrially viable and widely used method for producing NEs for total parenteral nutrition. It can be used to prepare NEs, SLNs and NLCs by rapidly pushing the pre-emulsion through a narrow channel. The most common problems, such as lipid crystallization (SLNs/NLCs) or drug degradation, could be avoided by closely monitoring production conditions (temperature and shear stress) [61]. Microfluidics, as opposed to the top-down technique of HPH, is one of the most convenient techniques for the continuous, bottom-up production of nanoparticles. This technique necessitates the use of two inlet streams, one containing lipids in a water-soluble solvent and the other an aqueous solution. As the streams flow in parallel, the mixing process begins, polarity shifts and lipid autoaggregation occurs in a reproducible manner. Depending on the chamber design, several techniques for producing lipid-based nanoparticles have been used, including T-junction mixing, hydrodynamic flow focusing and co-axial injection [62].

It is critical in the production of any DDS to select optimal formulation factors and process parameters that result in optimal values of critical quality attributes, such as size, polydispersity index or zeta potential. This can be realized using the quality by design (QbD) approach. QbD is defined as “an approach that aims to ensure the quality of medicines by employing statistical, analytical, and risk-management methodology in the design, development, and manufacturing of medicines” by the European Medicines Agency (EMA). Several analytical techniques and models have been used to improve formulation development in this vein [37,39,40,63,64]. The optimal design of experiments (DoE) should be chosen for the best results. Setting the objective (screening, optimization or prediction), selecting the factors and the range of their variation and selecting the type of design are all necessary steps. The influence of formulation factors such as stabilizer type and concentration, oil phase composition [21,35,37,40] or different process factors such as HPH pressure, temperature, the number of homogenization cycles, ultrasonication time, energy intensity and temperature [21,39,40] are chosen as independent variables, while the droplet/particle size, polydispersity index and zeta potential are usually chosen as dependent variables (responses). The DoE results in a model that describes the impact of the selected variables and/or their interactions, on the responses. Artificial neural networks (ANNs) are defined as parallel, distributed information processing structures used to model complex relationships between inputs and outputs or to find patterns in data. They are useful when a standard statistical analysis fails to recognize more complex, multi-dimensional and non-linear patterns [64]. The analytic hierarchy process (AHP), a multi-criteria decision-making tool, is another useful tool in formulation development. It is typically performed in the following steps: (a) structuring the hierarchical decision problem, (b) formation of the judgements matrix, based on pairwise comparison of criteria and alternatives, (c) consistency test that must be repeated until satisfactory results are obtained and (d) synthesizing comparisons across various levels to obtain the final weights of alternatives [63]. It can be used to compare different preparation techniques and select the one that results in the best formulation.

## 3. Selected Physicochemical Properties as Critical Quality Attributes of Nanomedicines

From the perspective of regulatory bodies, required pieces of information supporting the quality, safety and efficacy of any nanoenabled medicinal product are categorized into physicochemical and biological parameters [65]. Particle size and size distribution, shape, morphology, surface charge and other surface properties represent some of the critical quality attributes. This has been highlighted in one of the newest guidance documents issued by the FDA [66]. Moreover, size and size distribution are important regardless of the anticipated administration route and for all classes of nanostructured products. An interesting property of a nanomedicine is its inherent particulate character, leading to quite different biological interactions compared to a dissolved drug [67]. Therefore, their behavior and assessment of the same attributes in a biological (complex) environment is of crucial importance, due to direct relation to the safety and efficacy of nanomedicines [68].

Even though these properties are commonly assessed on the lab-scale level, applying a palette of techniques, specific methodological gaps still exist. This aspect is out of the scope of the article but is important to be considered when reporting results of sizing experiments. Reliable characterization protocols, adjusted to the complex structure of nanomedicines, are a must for their market success [65,69].

### 3.1. Size Estimation of Nanoparticles

Designing nanomedicines for brain targeting represents one of the most difficult tasks (due to the existence of BBB), even for receptor-mediated transport [70], and sizing experiments follow the nanomedicine candidate from preformulation studies to long-term stability evaluation. Interestingly, among more than 50 nanomedicines approved by the FDA so far, there are only several indicated for neurological disorders [71]. It has been reported that BBB overpass is size-dependent, and for a more successful brain disposition, nanoparticle size should be below 50 nm [72,73,74].

Many techniques are being used for size estimation: microscopy (electron microscopy (EM) and atomic force microscopy (AFM)), light scattering techniques (dynamic and static light scattering (SLS and DLS), laser diffraction (LD)), asymmetric flow field-flow-fractionation (AF4), centrifugation techniques (centrifugal liquid sedimentation (CLS), analytical ultracentrifugation (AUC)), tenable resisting pulse sensing, particle tracking analysis (PTA), etc. However, due to inherent differences in the working principles of the available techniques, different instruments may often provide different outputs (Table 2). Therefore, regulatory recommendations are encouraging the application of complementary methods, which could overcome these differences [66]. The orthogonal approach in the size estimation is commonly considered (measurements based on different physical principles to measure the same property of the same sample aiming to minimize the method-specific biases) [69]. Nonetheless, it is sometimes very difficult to compare the results of different techniques. Therefore, it is important to be knowledgeable on the working principles of each method. In addition, many of the stated methods are truly state-of-the art. However, lacking standardized procedures hampers the result comparison and reliability.

Based on a recent analysis [75], in most cases, stakeholders opted for DLS as a sizing technique. Even though it is standardized and user-friendly, it is considered a low-resolution technique, unable to distinct various particle populations in a polydisperse sample. Therefore it is not recommended as a unique sizing technique [76,77].

**Figure 2:**
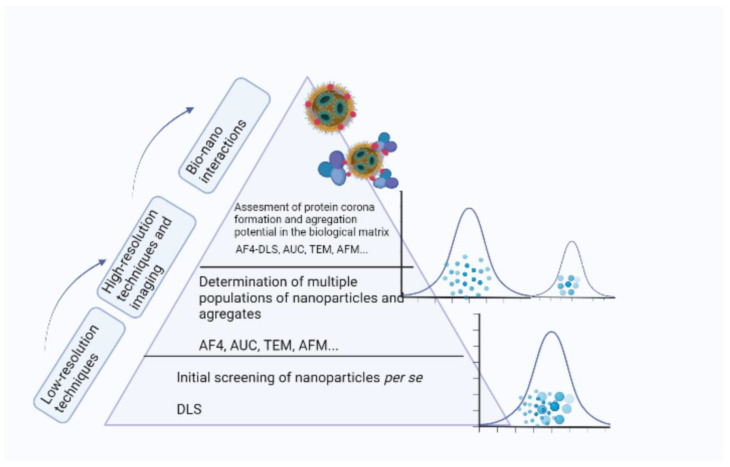
Necessary steps in the size characterization of nanoparticles (from bottom up): testing general sample properties applying low-resolution techniques; resolving multiple size populations of nanoparticles, aggregates, and larger particles; investigation on nanoparticle-protein interactions. Created with BioRender.com (accessed on 31 December 2022).

In order to address the problematics of proper nanoparticle size evaluation, leading scientific bodies in the field of nanomedicines have presented the so-called “three-step-approach“, with increasing complexity [68] (summarized in Figure 2):

1. Pre-screening (initial check which should indicate possible shortcomings before starting other, more demanding experiments) by a low-resolution technique (e.g., DLS);
2. More detailed analysis through one of the high-resolution techniques and appropriate microscopic analysis that provides visual inspection of particles;
3. The last step involves evaluating the size and potential for aggregation in the biological medium.

When choosing a technique for sizing and size distribution, it is necessary to take into account its suitability for a particular sample, possible interactions as well as the operating size range.

### 3.2. Protein Corona Formation

Upon entering blood circulation, nanomaterials interact with plasma proteins, resulting in the formation of a protein corona [66]. These interactions modify some nanoparticle properties, including size and cellular recognition, influencing safety and efficacy. Apart from size increase, protein corona may induce aggregation due to nanoparticle destabilization—as a more drastic event [68]. Stability assessment in complex media should be performed by applying high-resolution techniques [78].

The literature provides an overview of techniques that are appropriate for the estimation of some properties of the protein corona. They can be broadly divided into in situ (the ones that allow direct measurements of the nanomaterial in the biological medium) and ex situ (the ones that require nanomaterial isolation). Both approaches have their advantages and disadvantages. Even though the in-situ techniques are more relevant, they are limited in terms of the amount of information they could provide. On the other hand, nanomaterial isolation for ex situ techniques inevitably causes some structural changes in the protein corona and the loss of the loosely attached proteins [79]. Nevertheless, the structural assessment of the protein corona encompasses its thickness, density, protein identity and affinity towards the cells ([80], Table 3).

### 3.3. Nanoparticle Shape

Nanoparticle shape has been recognized as important from the aspect of the interaction of nanomaterials with the biological system and represents a necessary segment in the characterization. It is stated that slightly elongated (rod-shaped and ellipsoidal) shapes may be more successful for endocytose-mediated cellular uptake, probably due to the larger contact surface with the cell membrane. However, this is not a crucial feature if particles possess a specific surface functionalization (e.g., with receptor-specific ligands). The results of certain studies suggest that “soft” nanoparticles may have a better interaction with the cell membrane and thus be more successfully internalized into the cell [82]. Nanoparticle shape determination can be performed by microscopy techniques (EM, AFM), which also provide information on the particle size [68].

### 3.4. Surface Charge Estimation

Determination of the surface charge (actually, determination of the zeta-potential as a manner of its estimation) is a common characterization method, often with the aim to assess the kinetic stability of a nanodispersion, but also from the aspect of assessing bio-nano interactions [41,83].

Zeta potential can be determined by monitoring electrophoretic mobility of the nano-objects dispersed in an aqueous medium, using several methods described in ISO standards [84,85]. In routine laboratory practice, a method based on electrophoretic light scattering (ELS) is usually applied, which is then converted to zeta potential [77].

In general, higher absolute values of zeta potential are coupled with better long-term stability. In particular, if the dominant stabilization mechanism is electrostatic, the absolute values of zeta potentials above 30 mV are considered an indicator of good long-term stability [86,87]. However, these values cannot be taken strictly, especially when stabilization is provided by the use of steric stabilizers or a combination of electrical and steric effects. Due to the presence of steric stabilizers, during the measurement of the zeta potential, the diffuse layer does not move along with the particle in the electric field, so the shear plane is shifted, and consequently, zeta potential is measured at a greater distance from the Stern layer. Due to the exponential decay with an increase in the distance from the Stern layer, measured values are significantly lower compared to a system that does not contain steric stabilizers [88]. It is stated that, in the case of electro-steric stabilization, absolute values of zeta potential of about 20 mV are an indicator of good physical stability [89].

## 4. In Vitro Safety and Efficacy Aspects of Lipid-Based Nanoparticles

### 4.1. Evaluation of Endotoxin Presence

When evaluating in vitro safety, sterility is an important prerequisite, since LNs can be easily contaminated by bacteria or bacterial endotoxins (lipopolysaccharide, LPS) during production or handling. Although sterilization eliminates most biologically active contaminants, it is ineffective in eradicating heat-stable bacterial endotoxins. LPS can be easily bound to positively charged surfaces, thanks to its negative charge, but also to the lipid nanoparticles in general, by the hydrophobic interactions with the lipid domains [90]. Consequently, owing to potent inflammatory activity, the presence of endotoxins can generate misleading findings both in in vitro and in vivo assays aiming to evaluate the toxic and inflammatory effects of LNs, hindering the evaluation of their real biological effects [91]. Therefore, the presence of endotoxin in LNs has to be excluded before proceeding with other toxicity studies. However, although the impact of the sterilization process on physicochemical properties and stability of parenteral LNs has been frequently described in the literature, the endotoxin levels have been commonly overlooked. Currently, in order to evaluate the levels of endotoxin in nanomaterials, four methods are accepted by regulatory authorities, including the in vivo rabbit pyrogen test (RPT), in vitro Limulus amoebocyte lysate (LAL), the monocyte activation test (MAT) and recombinant factor C (rFC) assay [92]. Nevertheless, it should be kept in mind that LNs, due to specific physicochemical properties, have a strong propensity to interfere with the endotoxin contamination assessment (e.g., by adsorbing the assay components or by interfering with the final readout of the tests). The limitations of the conventional assays for LPS determination encouraged the development of several alternative approaches that have been successfully utilized in the literature for nanoparticle characterization. Different methods used to detect the endotoxin level in nanomaterials, their advantages and limitations, possible interferences of nanomaterials with endotoxin contamination assessment, as well as proposed approaches to overcome these issues have been detailed in several excellent reviews (cf. [90,92,93]).

### 4.2. In Vitro Evaluation of Hematological Compatibility

When designing lipid-based nanocarriers intended for parenteral administration, it is essential to thoroughly evaluate their compatibility with the blood system. After introduction into the bloodstream, nanoparticles may interact with the blood components, triggering numerous blood toxicities such as erythrocyte hemolysis, complement activation, perturbation of blood coagulation pathways, etc. Surface-related properties of LN, such as surface chemistry, surface coating and surface charge, generally display a crucial role in the interactions with the blood systems, but also other factors such as size, shape, elasticity and type of the incorporated drug can contribute to the blood toxicities of the LNs [94]. Hemolysis refers to the damage in the erythrocyte membrane, leading to the leakage of hemoglobin into the blood-stream. Therefore, the investigation of hemolytic activity is a highly useful test to assess the interactions of nanoparticles and cell membranes early, providing an initial insight into the biocompatibility of LNs. Basically, the in vitro hemolysis test enables us to study the degree of erythrocytes destruction after their exposure to test LN, by estimating the amount of hemoglobin released. In the presence of atmospheric oxygen, the released hemoglobin is converted to oxyhemoglobin which is further measured spectrophotometrically. In addition, usually, negative (sterile filtered PBS (pH 7.4) or 0.9% sodium chloride solution) and positive (1% Triton X-100 or 2% sodium dodecyl sulfate solution) controls are used as 0% and 100% of hemolysis, respectively. The percent of hemolysis is then calculated as [(A_nc_ − AL_N_)/(A_nc_ − A_pc_)] × 100; where A_nc_ represents absorbance of the negative control, AL_N_ absorbance for the tested LN, and A_pc_ absorbance of the positive control [95]. Although different hemolytic cutoffs, expressed as a percentage of in vitro hemolysis, were proposed, in general, <5% (or <10%) is considered non-hemolytic and >25% is considered hemolytic [96,97].

Analyzing the literature data regarding the in vitro hemolysis testing of parenteral LNs for brain targeting, quite different test protocols could be observed. Moreover, there are numerous inconsistencies regarding LN dose selection, blood preparation procedure (e.g., whole blood, diluted blood or erythrocyte suspension), species from which the blood was taken (e.g., human or rat blood), assay procedure (incubation time, centrifugation speed/time, determination of released hemoglobin) (e.g., [40,98,99,100,101,102]). Interestingly, most of the tested nanoemulsions, liposomes and solid lipid nanoparticles for the treatment of brain disorders have been described as non-hemolytic, under the experimental conditions employed, irrespective of the formulation composition and its physicochemical properties. However, it should be noted that only a limited number of studies reported the possible interference of nanoformulations with the spectrophotometric determination of the released hemoglobin. In order to avoid the interference of LN in absorbance readings, different approaches were proposed, such as the dilution of the samples after incubation with erythrocytes using dichloromethane [99] or ultracentrifugation [100]. In this regard, it is interesting to note that in September 2022, ASTM E2524-22, *Standard Test Method for Analysis of Hemolytic Properties of Nanoparticles* [103] was released, replacing E2524-08 (2013) that was withdrawn in April 2022. It is expected that this new standard will contribute to reducing the experimental variations and consequently, to obtaining more reliable data.

Upon entering the systemic circulation, apart from the erythrocytes, nanoparticles can also interact with the components of the coagulation system (primarily, platelets and plasma coagulation factors), leading to the perturbation of blood coagulation pathways (i.e., affecting the thrombostatic equilibrium) [104]. More precisely, in response to nanoparticles, platelet aggregation can occur due to the activation of the GPIIb/IIIa surface receptor (also known as integrin αIIbβ3) and the subsequent bridging of adjacent platelets by fibrinogen [105,106]. The absorption/binding of coagulation factors onto the nanoparticles’ surface (often found in the protein corona) may cause (i) inactivation/reduced availability of components of the coagulation cascade and subsequently, the prolongation in the coagulation process or (ii) activation of factors and undesirable coagulation [104]. Therefore, only a combination of in vitro assays for the evaluation of platelet aggregation and perturbation of plasma coagulation is considered promising to predict the procoagulant and anticoagulant properties in vivo [94,107]. For the in vitro analysis of platelet aggregation, the procedure recommended by Neun and Dobrovolskaia [107] was the most frequently used: platelet-rich plasma obtained from freshly derived human whole blood is incubated with the tested nanoformulation, positive (collagen) and negative (PBS or RPMI cell culture medium) controls; plasma is then examined using a particle count and size analyzer to measure the number of active platelets, whereas the percentage of aggregation is determined by the number of active platelets in the sample treated with the nanoparticle, relative to the applied controls. Similar to other discussed methodologies, a wide variety of test protocols were utilized in the literature to study platelet aggregation by parenteral LNs (e.g., [108,109,110]), limiting the comparison of the obtained data. For the evaluation of the nanoparticle-induced activation of the coagulation system, platelet-poor plasma from human whole blood should be exposed to the tested nanoformulation (or positive/negative control) in vitro, and analyzed using prothrombin (PT), activated partial thromboplastin (APTT) and thrombin time assays [107]. However, it is noteworthy that comprehensive studies dealing with the evaluation of thrombogenic potential of parenteral LNs intended for the treatment of CNS disorders are limited in number and scope. It was observed that liposomes designed for potential applications in the diagnosis and/or therapy of Alzheimer’s disease, and prepared with different combinations of five ligands (for brain/amyloid targeting), induced a slight to moderate decrease in the coagulation time, but without a statistically significant difference compared to the PEGylated liposomes with no targeting ligands on their surface. The effect of the developed liposomes on the platelet function was not monitored [100]. On the other hand, Koziara and coworkers [110] observed that non- and PEGylated cetyl alcohol/polysorbate nanoparticles did not activate the platelets in vitro, but moreover, inhibited the agonist-induced platelet activation and aggregation in a dose-dependent manner. However, at high concentrations, the tested nanoparticles significantly prolonged whole blood clotting time (analyzing visible signs of clot formation). Although the cancer drugs are out of the scope of this review, it is interesting to note that liposomes with a more negative charge and a larger size were able to induce significant complement activation, platelet aggregation and abnormal coagulation times compared to liposomes with lower surface charge density and smaller size [111]. Therefore, it is essential to ensure that the developed lipid nanoformulations do not affect the platelet function and coagulation cascade before proceeding to in vivo studies.

The complement activation is another important aspect that should be carefully examined when designing the lipid-based nanocarriers, particularly for the intravenous administration route. Analyzing the frequency of the blood incompatibilities of LNs, Urbán and coworkers [94] have observed that the complement activation was the main adversity of this nanoparticle type described in 39% of the reported studies. Among the lipid nanocarriers, intensive research has been mainly focused on the role of complement in the liposome stability and biological performance. The complement activation by liposomes was shown to have many consequences that limit the liposome-mediated targeted drug delivery. For example, the inclusion of the lytic C5b-9 complex into the bilayer was shown to provoke the leakage of encapsulated drugs from the liposomes [112,113]. Likewise, the binding of complement fragments C3b and iC3b to the surface of liposomes could increase their recognition by the macrophages with receptors for complements, circulating monocytes and neutrophils, thus limiting liposome targeting to the sites outside the reticuloendothelial system [113,114]. Furthermore, one of the most commonly reported clinical adverse effects after infusion of PEGylated liposomes (e.g., Doxil^®^, Taxol^®^) is a non-IgE-mediated hypersensitivity reaction (commonly called complement activation-related pseudo allergy (CARPA)), accompanied with various hemodynamic, respiratory, cutaneous and subjective manifestations. Considering that these symptoms usually occur at the first contact with the product (without previous sensitization), numerous pieces of evidence indicate that the complement activation, i.e., the rapid production of complement anaphylatoxins C3a and C5a and the subsequent release of thromboxane A2 and other anaphylatoxin-derived mediators, is involved in the pathogenesis of CARPA [112,114,115]. Intriguingly, Moghimi et al. [115] observed that the methylation of the phosphate oxygen moiety of phospholipid-methoxy (polyethylene glycol) conjugate omits the complement activation by conventional PEGylated liposomes, implying that a specific spatial organization of functional groups on the bilayer surface can substantially affect the biological fate of liposomes [111]. Likewise, liposomes modified with the hyaluronic acid, contrary to the PEGylated liposomes, did not trigger the complement activation in the human serum in vitro and rat plasma in vivo [116].

However, it is important to emphasize that it is quite difficult to test the complement activation in vivo, due to high interspecies variation and low predictability of hypersensitivity reactions in humans [117]. Currently, only the animal model based on pigs is considered suitable for the identification of immune reactive nanoparticles, though the oversensitivity limits its use [94,118]. Therefore, among different methods proposed for the analysis of complement activation (reviewed by Morales and Sims [119]), measuring the amounts of complement components iC3b and SC5b-9 by ELISA-based assay is most commonly used to evaluate the complement-related blood compatibility of endotoxin-free LNs [100]. Other complement components (such as C4a, C4d and Bb) can be determined in order to gain a deeper insight into the main pathway responsible for the complement activation by the tested nanoformulations [120]. It is essential to simultaneously include a negative control (usually PBS) and a positive control (e.g., cobra venom), to demonstrate the discriminatory power of the method. In more detail, an increase in the complement component species between 100 and 300% (over the value determined for the negative control) is considered as ‘an elevated risk’, while an increase higher than 300% was clinically relevant for the occurrence of hypersensitivity reactions in patients [100]. To the best of our knowledge, there are still no standardized assays for measuring the complement activation in vitro by nanoparticles. ASTM F1984-99(2018) [103] is predominantly intended for complement activation testing of medical devices that get in contact with the blood. As a result, a wide variety of experimental conditions were employed in different laboratories (e.g., blood preparation procedure, duration and storage conditions of serum/plasma, the extent of dilution of serum/plasma upon incubation, the conditions of incubation, source of ELISA kit) that can significantly affect the outcome of complement activation by liposomes and other lipid nanoparticles, leading often to inconsistent data (cf. [118]). Furthermore, due to significant biological variation in the serum level of complement proteins and various possible interactions, Moghimi and Hamad [112] suggested monitoring the liposome-mediated complement activation in fresh sera of at least five healthy individuals. In general, when properly designed, in vitro assays based on the blood obtained from healthy volunteers correlate well with the in vivo complement-mediated reactions, and therefore, can be highly useful in predicting biocompatibility of the developed nanoformulations [18,120].

### 4.3. In Vitro Cytotoxicity Evaluation

In vitro cytotoxicity testing is usually performed at the early stages of LN development to obtain the initial insight into the toxic potential of the designed formulation. When planning an experiment to study the cytotoxicity of nanoparticles, generally, several critical segments should be carefully addressed: (1) cell type selection—based on the intended administration route and the organ targeted by the nanoparticles, (2) the proper dose selection to test the toxic effect of nanoparticles—based on the anticipated concentration of nanoparticles and respective exposure; (3) selection of a suitable cytotoxicity assay—to avoid the interference and properly identify the toxic effect of the nanoparticles; (4) stability of nanoparticles in the biological environment [121]. The exposure of the brain to the nanoparticles can raise the risk of neurotoxicity [122], and thus the in vitro cytotoxicity studies in the neuronal cell lines should be performed [97]. Different studies dealing with the cytotoxicity evaluation of parenteral LNs for the treatment of CNS disorders are presented in Table 4. As can be seen, quite different cell types were employed, but generally, no remarkable cytotoxicity was observed for any tested lipid-based nanoformulation, irrespective of the formulation composition/targeting ligand(s) and physicochemical properties. Although cancer cells have been frequently used, it should be kept in mind that they exhibit different genetic and metabolic abnormalities, and thus cannot represent a realistic model for humans. Further, the proper selection of nanoparticle dose is crucial to evaluate the actual toxic effects of LNs, i.e., to avoid artificial toxicity induced by an unrealistically high dose [121]. Notably, the criteria for the selection of tested nanoparticle doses have rarely been provided in the aforementioned studies (Table 4). Commonly, the initial concentrations of LNs introduced into the cell culture medium are considered the effective concentrations. Far different from water-soluble compounds, nanoparticles, depending on the physicochemical properties, have a tendency to diffuse, agglomerate, aggregate and/or interact with the proteins of cell culture media. Subsequently, the number of nanoparticles in close contact with the cell monolayer during the assay can be significantly reduced. Therefore, in order to accurately evaluate the dose-response of nanoparticles, it is essential to determine the cellular dose [123]. Various methods have been proposed for the determination of cellular dose, such as UV–VIS absorbance measurements, light microscopy, mass spectrometry (MS), inductively coupled plasma MS (ICP-MS) and liquid chromatography MS (LC–MS) [121,123].

Additionally, 3-(4,5-dimethylthiazolyl-2)-2,5-diphenyltetrazolium bromide (MTT) reduction and lactate dehydrogenase (LDH) release tests are frequently used to study the toxicity of LNs targeting the brain disorders (Table 4). Interestingly, none of the presented studies reported potential interferences of the LNs with the selected assay. Namely, it is well known that nanoparticles can interact with the test reagents or at the assay readout, leading to false-positive or false-negative results [17,124]. Moreover, ISO/TR10993–22:2017 (E) [125], which provides a general framework for the biological evaluation of medical devices composed of or containing nanomaterials, highlights that various assays proposed are not always appropriate in the testing of nanomaterials. Therefore, it is highly recommended to use at least two assays based on different readouts (designed to run as separate or multiplex systems) to assess the impact of nanoparticles on cell viability. The appropriate controls, enabling us to assess the potential interactions of LNs and test reagents/the assay readout without the cells, should be involved [97]. Correspondingly, numerous other approaches have been proposed in the literature to overcome the interference of LNs with the cell-viability assays. For example, based on comparative results obtained during the in vitro cytotoxicity testing of liposomal preparation, the high content screening (HCS) method for monitoring tetramethylrhodamine methyl ester (TMRM) accumulations in mitochondria with intact membrane has been proposed as an alternative to colorimetric MTT assay. Similarly, it was shown that the HCS for assessing the cell viability via Hoechst/propidium iodide (PI) staining allows more accurate toxicity assessment compared to the LDH assay [17]. On other hand, Rösslein and coworkers [126] proposed the use of cause-and-effect (C&E) analysis to identify the sources of variability in 3-(4,5-dimethylthiazol-2-yl)-5-(3-carboxymethoxyphenyl)-2-(4-sulfophenyl)-2H-tetrazolium (MTS) cell-viability assay to design the robust, high-quality cell-based assay to test nanoparticle cytotoxicity. The authors proposed the usage of a 96-well plate layout which incorporates a range of control experiments to assess the multiple factors affecting the quality of the MTS assay system (such as nanomaterial interference, pipetting accuracy, cell maintenance, instrument performance, assay protocol, handling and characterization of the tested LN). Although the approach has been specifically applied for the MTS assay, it may be valid also to other toxicity assays after appropriate modifications. In other words, a thorough understanding of all contributors to the assay variability by the C&E analysis can ensure consistent performance, reproducibility and transferability of the assay [127].

**Table 4:** Examples of the studies investigating the in vitro cytotoxicity of lipid-based nanoparticles intended for the treatment of CNS disorders.

Type of LN	Active Substance	Cell Type	Viability Assay	Main Observations	Reference
SLN	Saquinavir	HBMECs cells	XTT assay	No effect on the cell viability of Tween 80 and P407 stabilized SLN. The inclusion of 83-14 MAb graft did not affect the cell viability.	[128]
SLN	Andrographolide	hCMEC/D3 cells	MTT assay LDH assay	Increased incubation time and dose of andrographolide-loaded SLN significantly decreased cell viability/increased cytotoxicity.	[129]
SLN	Methylprednisolone	Human glioblastoma-astrocytoma cells (U87MG)	MTT assay	PEGylated and SLN functionalized by anti-Contactin2 or anti-Neurofascin antibodies were less toxic compared to undecorated SLN.	[130]
SLN	/	hCMEC/D3 cells	PrestoBlue assay	No reduction in cell viability was observed in the presence of SLN functionalized by mApoE at any tested concentration, during 24 h incubation.	[131]
SLN	Astaxanthin	Primary olfactory ensheathing cells (OECs) Human breast cancer cells (MCF-7)	MTT assay	In normal cell line, no significant difference in the cell viability was observed between astaxanthin-loaded SLN and placebo. In cancer cell line, the significant reduction in cell viability was found for astaxanthin-loaded SLN.	[132]
SLN	Idebenone	Primary culture of astrocytes obtained from rat cerebral cortex	MTT assay LDH assay	The tested formulations differing in the type (cetheth-20/isoceteth-20) and portion of stabilizer did not affect the cell viability compared to the controls.	[133]
NLC	Riluzole	NSC-34 hCMEC/D3 cells	MTT assay	Riluzole-loaded NLC, including formulation functionalized with lactoferrin, did not induce substantial cytotoxic effect to the selected cell lines.	[5]
NLC	Curcumin	B.End3 cells RAW 264.7 macrophage cell line	MTT assay	In macrophages, the cell viability decreased with increasing the concentration of curcumin, whereby the cytotoxicity of plain NLC was higher compared to the polysorbate 80-based one.	[134]
NE	Valproic acid	hCMEC/D3 cells	MTT assay	Incorporation of valproic acid into the nanoemulsions significantly reduced its inherent cytotoxicity. NE formulation with higher oil content was less cytotoxic.	[55]
NE	Curcumin	Human lung fibroblast cells (MRC5)	MTT assay	Non- and PEGylated nanoemulsions had no significant effect on cell viability. An increase in formulation concentration slightly decreased cell viability, due to benzyl alcohol and its concentration-dependent cytotoxicity.	[40]
SLN NLC NE	/	Monkey kidney epithelial cells (VERO) Acute lymphoblastic leukemia cells (L1210)	MTT assay	The SLN formulation induced the higher cytotoxicity compared to NLC and NE formulations, implying that the lipid composition plays and important role in the cytotoxicity.	[99]
LP	Diosmetin	HepG2 cancer cells	Cell counting kit-8 (CCK-8) assay	Dose-dependent toxicity of LP was observed, with no significant difference between PEGylated LP functionalized with lactoferrin and free diosmetin.	[135]
LP	/	hCMEC/D3 cells	MTT assay	Mono- and dual-decorated LP, functionalized by OX26 mAb and/or peptide analogue of ApoE3 were non-toxic in selected cell line after 24 h incubation.	[136]
LP	Phosphatidic acid Cardiolipin	hCMEC/D3 cells neuroblastoma SH-SY5Y cells	MTT assay	The LP loaded with phosphatidic acid or cardiolipin did not affect the viability of both cell lines at the lipid concentrations tested.	[137]

Abbreviations: SLN—solid lipid nanoparticles; NE—nanoemulsions; LP—liposomes; NLC—nanostructured lipid carriers; P407—poloxamer 407; 83-14 MAb, an insulin-like peptidomimetic MAb with molecular weight of 150 kDa; human brain microvascular endothelial cells (HBMECs); hCMEC/D3 immortalized human brain capillary endothelial cells; OX26 mAb—anti-transferrin receptor monoclonal antibody; mApoE—human apolipoprotein E; NSC-34—hybrid cell line obtained by the fusion of motor neurons (from the spinal cords of mouse embryos with mouse neuroblastoma N18TG2 cells); B.End3 cells—the immortalized mouse brain endothelial cell line; /—the study was performed with a ‘placebo’ vehicle, without an actual active substance.

### 4.4. In Vitro Permeability across the BBB

**Figure 3:**
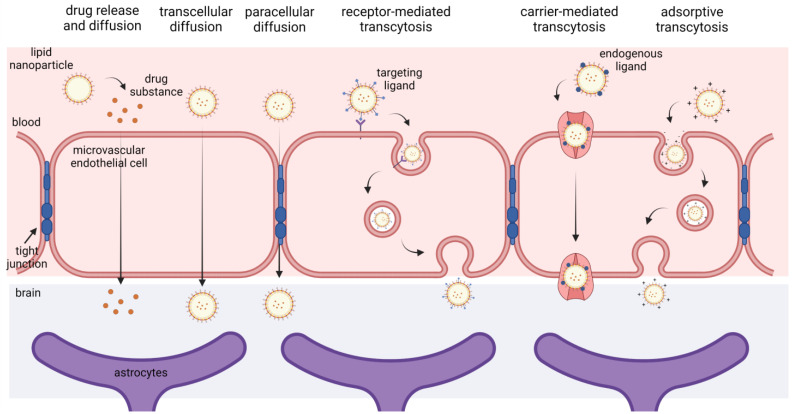
Proposed drug-delivery mechanisms across BBB in interaction with lipid nanoparticles [5,10,54,140]. Created with BioRender.com (accessed on 16 February 2022).

The in vitro models of the BBB are increasingly being used as valuable tools to screen the ability of various lipid-based nanoformulations to deliver drugs across the BBB, before the best candidate(s) are allowed to enter the pre-clinical in vivo animal studies. Considering the inherent biological properties of BBB, several mechanisms can be involved in the transport of drugs across the BBB using the LNs (Figure 3): (1) adsorption of the LNs onto the surface of brain capillary endothelial cells, providing a high concentration gradient for the diffusion of the released drug into the brain parenchyma, (2) transcytosis, endocytosis and exocytosis of LNs by brain capillary endothelial cells leading to direct penetration of intact LNs into the brain parenchyma, (3) transient BBB opening induced by stimuli derived from LNs (e.g., by the surfactants used for nanoparticle stabilization) resulting in diffusion of the released drug and/or drug-carrier conjugates into the brain parenchyma [138,139].

The majority of the in vitro BBB models employed for studying permeation of LNs targeting the CNS disorders (Table 5) are based on Transwell inserts. Moreover, most of the utilized BBB models enabled researchers to successfully discern differences in drug/nanoparticles passage across the BBB among the different formulations tested (Table 5). Basically, Transwell’s system represents a side-by-side vertical diffusion system which comprises ‘barrier’ cells grown on a microporous semipermeable membrane that divides two compartments representing the vascular and parenchymal side [141,142]. The simplest BBB model consists of a monolayer of endothelial cells in a Transwell insert. As can be seen in Table 5, commercially available immortalized human cell lines, such as the human cerebral microvascular endothelial cell line (hCMEC/D3), have been commonly employed for the creation of BBB. Although the immortalized cell lines can remain viable over many passages, offering higher experimental reproducibility between tests, they display a reduced ability to generate a tight monolayer with sufficient barrier function [143,144]. The inclusion of cyclic AMP and glucocorticoids has been shown to elevate the tightness and compensate for the deficiency of the endothelial monolayer [139,140]. Likewise, the addition of rat tail type I and/or type IV collagen and fibronectin to the semipermeable membrane can also improve the tightness and contribute to the development of a functional BBB [145]. On the other hand, primary brain endothelial cells enable the formation of the distinctly tight monolayer, with restrictive paracellular permeability, highly similar to the in vivo conditions. However, the BBB models based on primary cells can be used only for a short period of time (primary cells exhibit a pronounced tendency to dedifferentiate when subcultured), and simultaneously, are associated with high batch-to-batch variability and ethical constraints [145,146].

However, it should be emphasized that the monolayer BBB models based on one cell type generally do not adequately mimic the complex brain structural environment, due to the absence of barriergenic modulatory stimuli of adjacent cells [142,143,146]. Therefore, co-culture models, comprising brain endothelial cells, astrocytes (crucial for maintaining the barrier tightness) and/or pericytes (for inducing BBB differentiation and reducing pinocytosis) are considered more reliable in vitro BBB models suitable for the permeability studies [141,143]. Owing to relative simplicity, in vitro BBB models based on Transwell systems offer high-throughput screening of nanoparticles and relatively easy optimization of the experimental conditions [146]. However, there are several limitations of these systems that should be taken into consideration, including i) the two-dimensional architecture of the cells and ii) lack of physiological shear stress induced by blood flow. In other words, the Transwell systems lack the complexity of a neurovascular unit, which limits the reliability of their predictive value regarding the nanocarrier delivery into the brain [142,146,147]. Therefore, in order to overcome the shortcomings of 2D-BBB models and to better mimic the in vivo conditions, during recent years, intensive research efforts have been focused on the development and validation of dynamic 3D in vitro models and microfluidics-based BBB-on-chip models. The main features of these models have been reviewed recently (cf. [143,148]). However, it should be emphasized that 3D BBB models have been poorly employed to predict the BBB permeability of LNs. In this regard, it is interesting to note that Papademetriou and coworkers [149] showed, using the microfluidic-based BBB model, that flow significantly modulates binding to the endothelial cells and BBB penetration of Angiopep-2 functionalized liposomes, highlighting the importance of the local flow environment in the in vitro BBB models.

As laid out in Table 5, when evaluating the permeation of LNs through the BBB in vitro, numerous additional assays and technologies are needed, including the methods for the assessment of BBB properties (such as tightness, integrity and permeability). The transepithelial/transendothelial electrical resistance (TEER) is a commonly employed method to measure the integrity of tight junction dynamics in a monolayer cell culture [150]. In addition, in order to prove the barrier integrity, the paracellular permeability should be also assessed, by adding the fluorescent tracer substances when the steady-state TEER has been achieved and by the periodical determination of its concentration in the basolateral compartment [143,146]. In addition, apart from the restrictive properties, it is essential to evaluate whether all endothelial transporters are functioning correctly (e.g., efflux pumps to avoid the overestimation of nanoparticle transport into the brain). This is frequently overlooked when developing the in vitro BBB models and consequently, the role of the nanoparticles’ active transport into and out of the brain cannot be fully understood [147]. However, there are also opposite examples—modified non-cerebral cell lines (e.g., Madin-Darby canine kidney cell line transfected with the human MDR1 gene that encodes the polarized expression P-glycoprotein) have been used to screen substrates or inhibitors of efflux pumps at the BBB, and to evaluate whether the pump blockage contributed to the enhanced brain delivery of nanoparticles [145,151]. Hence, considering that each currently available in vitro BBB model displays certain limitations, there is a need for a comprehensive model which incorporates easy fabrication, ease of characterization, validation and testing for various parameters, simultaneously allowing the realistic simulation of LNs passage across the BBB.

**Table 5:** Exemplary studies that utilized various in vitro BBB models during the development of parenteral lipid-based nanoparticles developed for the treatment of CNS disorders.

Type of LN	Active Substance	In Vitro BBB Model	Methods Used for BBB Integrity Assessment	Main Observations	Reference
SLN	Saquinavir	Transwell system Co-culture of HBMECs and human astrocytes	TEER measurement	Tween 80 and P407 significantly improved SLN permeability across the BBB compared to SDS. The graft of 83-14 Mab significantly promoted SLN delivery across the BBB, particularly in combination with P407 and Tween 80.	[128]
SLN	Idebenone	Transwell system Madin-Darby canine kidney (MDCKII-MDR1) cells	TEER measurement	Idebenone permeability across the BBB from SLN was 0.4–0.5 lower than free drug (release was the rate-limiting step). It was suggested that idebenone permeates through the BBB predominately via a transcellular pathway.	[151]
SLN	Donepezil	Transwell system Triple co-culture of primary rat brain endothelial cells, pericytes and astrocytes	TEER measurement Permeability evaluation: flux of sodium fluorescein and Evans blue-labeled serum albumin	SLN functionalized with ApoE targeting ligand have led to a 3.2-fold increase in permeability of donepezil compared to non-targeted SLN.	[152]
SLN	/	Transwell system hCMEC/D3	Permeability evaluation: flux of paracellular (Lucifer yellow) and transcellular (propranolol) markers.	Functionalized SLN-Palmitate-ApoE and SLN-DSPE-ApoE significantly enhanced transport across BBB compared to the non-functionalized SLN. The tested nanoparticles could permeate the BBB predominantly via a transcellular route.	[153]
SLN	Resveratrol and Grape extract	Transwell system Co-culture of endothelial cells derived from hematopoietic stem cells and astrocytes	Permeability evaluation: radiolabeled ^14^C-sucrose	Transport of SLN functionalized with OX26 mAb was 2-fold higher than the SLN functionalized with a protein non-specific for BBB that recognizes α-synuclein (mAb LB509) and 4-fold higher than SLN alone.	[154]
NE	Valproic acid	Transwell system Co-culture of hCMEC/D3 and normal human astrocytes (CC-2565)	TEER measurements	No differences in the apparent permeability of valproic acid from developed nanoemulsions and valproic acid solution was observed (no correlation with in vivo animal study).	[55]
LP	Andrographolide	Transwell system hCMEC/D3	Permeability evaluation: flux of sodium fluorescein Integrity: phase-contrast microscopy or under bright-field optics s	The amount of andrographolide permeated across the BBB from LPs was 200-fold higher compared to free molecules. No remarkable difference was found between LP prepared with Tween 80 and cationic LP with Tween 80 and DDAB.	[32]
LP	/	Transwell system hCMEC/D3	TEER measurement Integrity of monolayer: microscope Permeability: flux of Lucifer yellow	The transport across the BBB of LPs functionalized by OX-26 mAb was higher compared to controls (PEGylated liposome and mouse IgG-immunoliposomes). The obtained findings indicated the lysosomal localization and receptor-mediated permeation with minimal paracellular delivery.	[136]
LP	/	Microfluidic system bEnd.3 cells	TEER measurements Immunofluorescence imaging of tight junction proteins	The flow within the microfluidic device significantly affected the binding and BBB permeation of Angiopep-2-functionalized nanoparticles, emphasizing the importance of flow environment for in vitro modeling of nanoparticle permeation through the BBB.	[149]

Abbreviations: SLN—solid lipid nanoparticles; NE—nanoemulsions; LP—liposomes; human brain microvascular endothelial cells (HBMECs); P407—poloxamer 407; 83-14 MAb—an insulin-like peptidomimetic MAb with molecular weight of 150 kDa, hCMEC/D3 immortalized human brain capillary endothelial cells; DDAB—didecyldimethylammonium bromide; OX26 mAb—anti-transferrin receptor monoclonal antibody; bEnd.3 cells—a polyoma middle T-transformed mouse brain EC cell line; /—the study was performed with a ‘placebo’ vehicle, without an actual active substance.

## 5. In Vivo Pharmacokinetic and Biodistribution Studies

**Figure 4:**
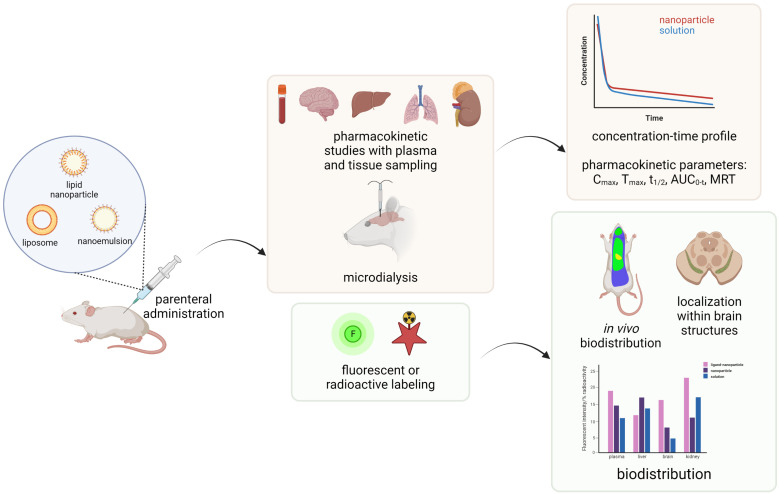
Different methodologies used in pharmacokinetic and biodistribution studies of lipid nanocarriers. Created with BioRender.com (accessed on 29 December 2022).

When analyzing drug delivery over some kind of barrier, two main aspects, the rate and the extent, are being considered. In the case of brain delivery, the rate is more important for drugs with fast effects, such as anesthetics and analgesics. For other pharmacological groups of drugs, which are administered repeatedly, the extent of the brain delivery is more significant, as it will often determine the therapeutic response. It is mostly characterized by transport across the BBB. Parameters describing the rate and the extent of the transport are determined in neuropharmacokinetic studies. They describe the fate in the brain of a drug, including its distribution and elimination [155]. As previously mentioned, when the drug is incorporated in nanoparticles, its pharmacokinetics is influenced by the formulation, i.e., the interaction between the carrier and plasma proteins or transporters, leading to alterations in distribution and clearance from the body [156]. Therefore, in most studies, pharmacokinetics and biodistribution of the nanocarrier intended for brain drug delivery are used as proof of concept of their successful development. However, different methodologies (Figure 4) are used throughout the literature, depending on the desired outcome and the set hypothesis, all with different advantages and disadvantages. In this review, we aimed to systematically evaluate different in vivo pharmacokinetic and biodistribution methods following parenteral administration of different types of lipid nanocarriers, and pointing out their strengths and limitations.

### 5.1. Pharmacokinetic Studies with Plasma and Tissue Sampling

Depending on the experimental setup, pharmacokinetic studies can be used (1) as a proof of concept of the drug’s brain delivery by nanoparticulate formulations, (2) to estimate the potential of different formulations for brain delivery, (3) to investigate the biodistribution to brain and other organs, and (4) to prove the passage into the brain parenchyma. In most in vivo studies, classical pharmacokinetic experiment with blood sampling is combined with the brain uptake study. In rodents, repetitive blood sampling is most commonly performed from saphenous, femoral or jugular vein, or orbit venous plexus. Brain uptake is then evaluated in a separate study after decapitation. While the plasma pharmacokinetic analysis is established based on many sampling time points, for the brain uptake study, in the majority of published studies tissue sampling was done in one to three time points. Still, Kozlovskaya and Stepensky [157] recommended conducting a detailed analysis based on the concentration data in the complete time course of the drug in the brain, blood and other tissues. In some of the reviewed studies, blood is collected via cardiac puncture from the same animals used for the brain sampling. Being a terminal experiment, one of the challenges in the investigation of brain pharmacokinetics is high variability of results. In order to estimate the area under the curve (AUC) in the brain and plasma it is necessary to perform the tissue sampling in five to six time points, which implies the use of a large number of experimental animals. In an attempt to reduce the number of animals in these studies, the number of time points for brain sampling may be reduced to three or even one. However, if the kinetics is unknown, e.g., if the concentration profiles in blood and brain are not parallel, the analysis and conclusions derived from the compared parameters could be erroneous [155].

Diverse protocols of tissue processing are described in the literature. While blood processing is more or less consistent through various laboratories, the processing of the brain tissue is rather different. In some cases, the unprocessed brain is used (after it was dried with paper to remove the excess blood), sometimes it is only rinsed with purified water, phosphate buffer or saline, and in some occasions, the blood from the brain is first removed by perfusion. After homogenization, the drug concentration in the brain is determined by a validated HPLC or mass spectrometry method. Further calculation of pharmacokinetic parameters is done using the non-compartmental analysis, by different software. Main pharmacokinetic parameters include maximum concentration (C_max_), time to reach maximum concentration (T_max_), area under the concentration versus time curve from zero to the last measurable time point (AUC_0–t_), and terminal elimination half-life (t_1/2_).

Different parameters are used for the quantification of brain-targeting efficacy. One of the parameters, partition coefficient (Kp), was used in research with diazepam nanoemulsions to differentiate the efficacy of the brain targeting among three formulations with different volumes of the oil phase [158]. The partition coefficient is the ratio between AUC in brain (or any other targeting organ) and AUC in plasma. Instead of using AUC values, this can be also calculated from the concentration data for each time point. This parameter is also known as the brain-targeting index. The higher the index, the more effective delivery is into the brain. However, in the extensive analysis of the quantitative aspects of brain drug delivery using different types and subtypes of nanodelivery systems [157], it was questionable whether the values above 10 could be attained considering the relative brain weight, without substantial disruption of the BBB. These high values would raise questions regarding the safety of the applied carriers. The assessment of the brain-targeting ability of different formulations could be done through the calculation of the brain enhancement factor. It represents the ratio between the concentration or AUC in the brain achieved with a nanoformulation and AUC after another comparative formulation (dispersion, solution, etc.). This parameter was used in experiments with carbamazepine NLC to compare the developed nanocarrier to the dispersion. It was found to be in a range of 1.35 to 5.00, depending on the time point, suggesting enhanced brain accumulation of carbamazepine, but also different distribution and elimination kinetics of the two formulations [159].

In most studies, nanocarriers influence the biodistribution of the drug. As a result, distribution to the target organ may be delayed. For example, after intraperitoneal administration of risperidon nanoemulsions, different patterns of absorption and distribution to the brain of the developed nanoformulations were observed. For two nanoemulsions, stabilized by poloxamer 188 or polysorbate 80, a longer time was needed to reach the maximum concentration in the brain. Moreover, elevated concentrations were obtained in the last time point following the administration of the mentioned formulations, suggesting a time-consuming process behind the brain uptake, such as receptor-mediated endocytosis or transcytosis [21]. In the next study by the same research group, the concentrations of risperidon and its active metabolite were determined in plasma, brain and liver. After nanoemulsions’ administration, higher AUC, longer mean residence time (MRT) and reduced clearance was obtained in plasma, together with the increased AUC in the brain, while in the liver AUC and MRT were reduced, in comparison to the solution. In this vein, the elevated levels in the brain could be the consequence of the higher brain penetration as well as decreased liver uptake [160].

Another interesting example of extended drug circulation is the pharmacokinetics of breviscapine when incorporated in SLN. When administered as a solution, scutellarin’s (active component of breviscapine) initial concentration in plasma was quickly reduced due to its fast elimination; however, when incorporated into the SLN the circulation was prolonged. At the start of the tissue distribution, the concentration in plasma rapidly decreased. The distribution to the brain was enhanced with a higher fraction of the plasma concentration that reached the target organ. The uptake was probably facilitated through the synergistic effects of stabilizers on the nanoparticle surface because PEG derivatives acted as inhibitors of the P-glycoprotein efflux pumps [161].

One of the main mechanisms of effective brain delivery of drugs using nanocarriers is considered to be the improvement of the permeability through BBB. However, in many pharmacokinetic studies, it was shown that the increased brain bioavailability was actually a consequence of the longer circulation time. For example, in a pharmacokinetic study of valproic acid nanoemulsions, the improved bioavailability in brain characterized by high brain partition coefficient was the consequence of the prolonged half-life in plasma. Results from the in vitro BBB cell culture experiments revealed not significantly different permeability of the drug when valproic acid was incorporated in nanoemulsion compared to the free drug in the solution [55]. However, in the mentioned in vitro experiment, the interaction of the NE stabilizer (polysorbate 80) and apolipoproteinE, which may facilitate the NE’s passage to the brain, was not taken into account, leaving the possibility of the active uptake of NE’s droplets into the brain in vivo.

Surface coverage of nanocarriers often determines their interaction with proteins and transporters. It is well known that the opsonization of nanoparticulate systems can happen fast upon parenteral administration. This leads to their uptake by the elements of the mononuclear phagocyte system and relatively fast clearance from the body [5]. However, in many studies, long circulation of nanoparticles was observed, when compared to the free drug substance (solution). Longer t_1/2_ and MRT in plasma often indicate improved metabolic stability and more time available for the interaction with transporters and passage through BBB. Even though lipid nanocarriers are designed to enable brain delivery, the majority of them are developed for the passive targeting. Therefore, a high percent of the administered dose often gets accumulated in the organs of mononuclear phagocyte system (liver and spleen), lungs and kidneys. Actually, drug concentrations in these organs usually exceeded the ones in the brain [157]. Active targeting, in turn, could provide bypassing of the peripheral organs. This was achieved with liposomes of dopamine, functionalized with a peptide of five amino acids from the amyloid precursor protein (APP). This ligand is recognized by specific transporters in the BBB, enabling the delivery into the brain [30].

The efficacy of the transport to the brain parenchyma is particularly important aspect that should be dealt with in pharmacokinetic studies. In order to estimate the drug concentration in the brain parenchyma, many researchers correct the data obtained from the bulk brain with the blood volume in brain vessels. However, in such a study design, the contribution of nanoparticles adsorbed to the luminal wall of microvascular endothelial cells, and cellular and membrane components of BBB is not taken into the account [162]. The mentioned correction was done in a study with lipid nanoparticles (SLN and NLC) of curcumin. The in vivo data revealed that the ratio between brain and plasma concentration was increasing in the first hour of the experiment. This was explained by the enhanced transport from plasma to the brain during this time and passage from the brain to plasma afterwards. There was a linear correlation between plasma AUC0-t/plasma concentration at time point t, and brain/plasma concentration at time point t. While the slope of the plot represented the brain uptake clearance, the intercept was considered as an estimation of the volume of the brain occupied by the plasma circulating in brain microvasculature. The bigger intercept meant higher levels of curcumin present in the brain tissue [163].

A more precise method for the estimation of the drug levels in brain parenchyma is its complete separation from the brain microvessels. The capillary depletion method, often combined with in situ brain perfusion, is used to separate endothelial cells from the brain cells and extracellular fluid. In this way, it could be determined if nanoparticles crossed the BBB or remained associated with the microvascular endothelial cells [164]. This method is based on centrifugal separation after homogenization and the addition of dextran. The supernatant is the brain parenchymal fraction, and the pellet is the capillary fraction. It was used in the investigation of lipid nanoparticles containing α-asarone. Aside from the prolonged residence time in plasma when compared to the solution, lipid nanoparticles enabled better BBB permeability leading to higher brain bioavailability. More importantly, it was proved that most α-asarone penetrated into the brain parenchyma [165].

### 5.2. Microdialysis

Microdialysis is another method for the analysis of nanoparticles passage to the brain. It was proven to be an important method to estimate unbound concentrations in tissues, especially the brain, and their association with pharmacological effects [166]. However, this method also has some limitations: due to too big size, nanoparticles would not diffuse through microdialysis membrane, while the appearance of nanoparticle components in the dialysate from a probe would not necessarily demonstrate its passage into the brain parenchyma. Therefore, the passage of intact nanoparticles to the brain is not likely to be determined by this method. When BBB is intact, it is assumed that microdialysis samples the brain’s extracellular space [162]. Brain microdialysis implies having a probe comprised of tubings and a semi-permeable membrane placed in the tissue. Perfusion fluid (perfusate) is pumped at a constant flow through the probe and solutes diffuse into the probe driven by passive diffusion. Dissolved substances are sampled at the end of the outlet tubing (dialysate), outside of the animal. The usual cut-off of the membrane is between 6 and 100 kDa. The exchange across the semi-permeable membrane will reach different degrees of equilibrium depending on the flow rate of the buffer. Therefore, using higher flow rates, in case of a need for more frequent sampling, can cause lower recovery across the probe membrane. For the quantitative measurements, measuring recovery in vivo is required [166].

Microdialysis was utilized to determine the brain disposition of melatonin when incorporated in liposomes or nanoemulsions, compared to the solution. In a pharmacokinetic study in plasma, surprisingly, lower concentrations, AUC and t1/2 were observed for the developed nanoformulations. On the contrary, in the brain, melatonin levels after both formulations were superior to the solution. This could be the consequence of the fusion of nanocarriers to the endothelial luminal membrane rather than the passage of intact nanoparticles through BBB. After the fusion, melatonin would be released in brain endothelial cells and afterwards transported to the brain tissue. Additionally, it was concluded that real melatonin concentrations in the extracellular fluid could be even higher than the ones observed due to the lower rate of analyte replacement to the probe membrane surface than of its removal from the inside of the probe [167].

In another very detailed pharmacokinetic investigation, microdialysis was used to determine the unbound brain and plasma concentrations of quetiapine after the intravenous administration of lipid-core nanocapsules or solutions. Plasma microdialysis was conducted to evaluate the protein binding when quetiapine was administered encapsulated in nanoparticles. Additionally, in order to estimate the influence of nanoencapsulation on the quetiapine brain penetration, probenecid solution was administered prior the quetiapine treatments. Probenecid is a drug-transporter inhibitor in BBB, liver and kidneys, so the co-administration of probenecid and quetiapine solution resulted in increased plasma exposure, but decreased distribution to tissues. Compared to the free form, nanoparticles reduced clearance from the body, due to the sustained release that was confirmed by the analysis of the unbound plasma profile. As mentioned earlier, nanoparticles do not cross the microdialysis membrane, meaning that the drug concentration in the dialysate is associated only to the free levels released from the nanocarrier in the tissue. It was deduced that quetiapine was circulating in the bloodstream and loaded in the nanoparticles for up to two hours. This strong attachment to the carrier disabled the interaction of the drug with the influx transporters, which led to lower penetration to tissues. Therefore, the transport to the brain was not influenced by probenecid, when quetiapine was administered in the encapsulated form, proving that the drug reached the brain parenchyma carried by the nanoparticle, while the BBB permeability was not altered [140].

### 5.3. Fluorescent and Radioactive Labeling

Bio-imaging techniques comprise non-invasive methods to visualize biological tissues in real time. They can provide an overview of the organ of interest or the whole body. Most commonly, they are combined with pharmacokinetic studies in order to evaluate the biodistribution of nanoparticles not only to the target organ but to other tissues such as the liver and spleen as well. Usually, this is done by fluorescent imaging with different dyes loaded in nanoparticles such as fluorescein isothiocynate (FITC), coumarin 6 or DiR in real-time near-infrared (NIR) fluorescence imaging. NIR has high spatial resolution, thorough molecular tracking of fluorescent probes and offers significant real-time display. Usually the imaging is done at early time points after the administration (2, 6, 12 and 24 h) [19]. This methodology was used post-mortem in a study of α-mangostin liposomes. The pharmacokinetic study revealed that the highest levels of α-mangostin in the brain were achieved when it was administered in transferrin-modified liposomes compared to the unmodified liposomes and solution, while fluorescent imaging gave more detailed localization within the brain–cerebellum and brainstem. The overall pharmacokinetic profile in the brain was improved due to the sustained effect of liposomes [168]. In a study with dopamine derivate, RVG-29-liposomes were more efficient for brain delivery compared to PEG-liposomes and solution. Bio-imaging was used for the investigation of the biodistribution of the developed nanocarriers. Mice were anaesthetized and imaged with an in vivo imaging system, which showed an accumulation of nanoparticles in the brain but also in the liver and spleen. By fluorescent microscopy the accumulation of the developed liposomes in striatum and substantia nigra, as targeted structures in the brain, was confirmed [54].

Alternative methodology with fluorescent probes is based on the evaluation of the fluorescence intensity values normalized to the organ weight. Similarly, as in pharmacokinetic studies, blood and different organs are collected at predetermined time points after administration and the fluorescent emission is recorded by a microplate reader. The ability of the nanocarrier to deliver fluorescent dye after intraperitoneal administration was confirmed in research with monoolein nanoparticles, stabilized by polysorbate 80. The fluorescence signal of coumarin-6 was monitored in the brain and liver, but it was not established whether the dye was detected in the brain tissue as a free substance or attached to nanoparticles. Nevertheless, the dye was delivered three hours after the treatment [169] which may suggest the complex mechanism behind the transport and stress the importance of the longer circulation time of the nanoparticles.

Fluorescent microscopy could complement brain uptake study with more information about the localization within the tissue. The fluorescent dye (rhodamine-123, FITC or coumarin-6) is loaded or attached to the nanoparticles prior to administration, while the fluorescent dye solution usually serves as a comparative formulation [19]. Additionally, the administration of the vehicle is included in the study as a control in order to exclude a possible autofluorescence of the tissue. At the time of sacrifice, the animal is perfused transcardially (usually firstly with saline solution) to remove blood components and afterwards with ice-cold paraformaldehyde in PBS under deep anesthesia. Coronal sections of the collected brain or its parts are then analyzed by fluorescent microscopy. This approach was used in a detailed investigation of the andrographolide distribution to the brain by Graverini et al. [129] using fluorescent microscopy. After intravenous administration, brain delivery was delayed due to the longer circulation. A high level of SLN was present in the brain, 24 h after the treatment, but after 3 days they were probably degraded. Nanoparticles were found in blood vessels as well as in brain parenchyma, while the free fluorescent dye was retained in blood vessels, clearly demonstrating the ability of the nanoparticles to cross the BBB. Furthermore, according to the absence of microglia activation, developed nanoparticles were not recognized as foreign bodies. Nevertheless, the exact mechanism of the passage to the brain was not explained.

In the above-mentioned study, microglia activation was analyzed by immunohistochemistry. This method is considered the gold-standard for the histological recognition of different cell types. It is based on the specific interactions between antibodies and antigens expressed on certain cell types, therefore acting as distinctive identifiers. The antibodies are labeled with fluorophores, which allows imaging by the fluorescent microscope. The protocol consists of the incubation of the cross-section of the brain with the antigen-specific antibodies, and afterwards the incubation with the fluorescent secondary antibody. After that, the samples are rinsed and prepared for the analysis [19].

Except for fluorolabeling, radiolabeling of the nanoparticles is applied for the determination of their in vivo pharmacokinetics and biodistribution, and especially brain distribution. In a study by Kakkar et al. [170] (2013), 99mTc was used as a radionuclide. It represents a short-lived metastable isotope of technetium (half-life around 6 h), with a γ photon emission energy suitable for imaging [171]. However, the labeling efficiency has to be determined prior to administration by an appropriate method. Such a study was done with the curcumin-loaded SLN. After the tissue collection, the nanoparticle uptake was evaluated by a gamma counter and the extent of distribution was expressed as % radioactivity per gram of the organ. Biodistribution studies can also be performed with 99mTc-labeled nanocarriers conducting the gamma scintigraphy of the animal’s body after administration. Aside from the 30-times higher AUC in the brain after intravenous SLN administration, significant radioactivity was noticed in the liver and lungs as organs with high blood supply, irrespective of the treatment. Still, the distribution to the liver was lower when curcumin was encapsulated in SLN, which is significant considering its excessive metabolism [170].

## 6. Concluding Remarks

The present review aimed to elucidate the current status and perspectives of parenteral application of lipid-based nanoplatforms in CNS disorders. Patients with these predominantly chronic disorders not only need protracted treatment with preferably oral administration of pharmaceuticals, but also a reliably fast and accurate brain exposure in more acute circumstances, which optimally may be accomplished by the parenteral administration. Due to the demanding physicochemical properties of pharmaceuticals with primary CNS activity, and the need to diminish off-target exposure by targeted disposition, parenteral lipid-based nanosystems seem to be the best theoretical fit for such a goal. Although with an apparently wide coverage, this niche has surprisingly few clinically proven outcomes. First of all, injectable emulsion with the general anesthetic propofol, widely used in anesthesiology, is arguably the first nanomedicine on the global market (cf. [71]). The use of other CNS nanomedicines, such as nanoemulsion with diazepam, is much scarcer. A recent program of development of glutathione-conjugated nanostructured formulation with methylprednisolone, aimed to favor brain exposure in relapses of multiple sclerosis thanks to recognition by glutathione transporters on the blood–brain barrier, generated encouraging results in clinical trials, but the pharmaceutical has not yet reached the market [172].

As a peculiar feature, the characterization of nanomedicines can pursue different levels of complexity. The nanomedicine-specific analytical challenges have a profound impact on the applicability of these medicines in the clinical setting. The European Commission has acknowledged the lack of established robust and *fit-to-purpose* methods to provide reliable preclinical data represents the bottleneck in the process of bringing promising nanomedicines to the market—regardless of the target site [173]. This has evoked the interest of the regulatory stakeholders, supporting further development of certain more sophisticated techniques. As indicated in the recent overview of the topic, prepared by Halamoda-Kenzaoui et al. [65], even though some methods have been standardized for the assessment of certain critical quality attributes of the nanomedicines, there is still a long way to go. Uncertainty in the nanomedicine field is multifactorial and reflected in the lack of clarity in in vitro—in vivo correlation, with many unknowns about details in assessing solubility enhancement, alterations in biodistribution, safety aspects and, in the context of brain targeting, evaluation of the blood-brain barrier behavior.

Therefore, this article summarizes some established methods, in parallel with the newest strategies in the preclinical characterization of nanomedicines for specific CNS disorders, pointing out some issues in the field, with an idea of reducing pitfalls. Potentially, it will raise more discussion on the necessary incremental improvement of the methods described, which could be a path to support the transfer of CNS drug carriers into a clinical trial. The lack of a universal fitting strategy is exemplified by liposomes, which have not fully met some initially set expectations in clinical practice, primarily in the context of pharmacokinetics and expected therapeutic outcomes in oncology [174]. In our opinion, nanostructured lipid carriers offer an additional opportunity to efficiently target brain tissue—not only after parenteral, but also oral administration (cf. [175] in press).

## Figures and Tables

**Table 2:** Some of the commonly applied techniques in size measurements, corresponding working principles and the type of determined size.

Technique	Physical Principle	Output
Static light scattering	Anisotropic light scattering	Diameter of gyration
Dynamic light scattering	Brownian motion	Hydrodynamic diameter
Particle-tracking analysis	Brownian motion	Hydrodynamic diameter
Tunable-resisting pulse sensing	Change in the ionic current	Hydrodynamic diameter
Analytical ultracentrifugation	Changes in particle concentration profiles during centrifugation	Hydrodynamic diameter
Centrifugal particle sedimentation	Particle sedimentation versus time	Hydrodynamic diameter
Atomic force microscopy	Atomic force between a probe tip and a particle	Geometric diameter (and particle visualization)
Scanning electron microscopy	Electron contrast in a scanning mode	Diameter of an equivalent sphere (and particle visualization)
Transmission electron microscopy	Electron contrast in a transmission mode	Diameter of an equivalent sphere (and particle visualization)
Field-Flow Fractionation	Separation achieved through the interaction of nanomaterials with an external physical field	Detector dependent

**Table 3:** Selected protein corona parameters and methods for their assessment [81].

Parameter of the Protein Corona	In Situ Techniques	Ex Situ Techniques
Thickness	Dynamic light scattering Fluorescence correlation spectrometry Small-angle X-ray diffraction	Differential centrifugal sedimentation Size exclusion chromatography Transmission electron microscopy
Density of the adsorbed proteins		Colourimetric protein assays
Identity and quantity of the adsorbed proteins		Poly(acrylamide) gel electrophoresis Liquid chromatography tandem mass spectrometry
Protein conformation		Circular dichroism Fluorescence quenching
Affinity		Size exclusion chromatography Surface plasmon resonance Isothermal titration calorimetry

## Data Availability

Not applicable.
